# 2,4-Dibromo-6-*tert*-butyl­benzene-1,3-diol

**DOI:** 10.1107/S1600536811034866

**Published:** 2011-09-14

**Authors:** Xia Wang, Jun-long Niu, Cai-xia Zhi

**Affiliations:** a Pharmacy College, Henan University of Traditional Chinese Medicine, Zhengzhou 450008, People’s Republic of China; b Department of Chemistry, Henan Key Laboratory of Chemical Biology and Organic Chemistry, Zhengzhou University, Zhengzhou 450052, People’s Republic of China

## Abstract

In the title compound, C_10_H_12_Br_2_O_2_, a multiply substituted bromo­arene, the C—C—C angles within the aromatic ring are in the range 115.7 (7)-122.4 (7)°. In the crystal, mol­ecules are linked by O—H⋯O hydrogen bonds, but no π–π stacking is observed.

## Related literature

For similar compounds, see: Butler & Walker (1993 ▶); Seevers & Counsell (1982 ▶); Zheng *et al.* (2004 ▶).
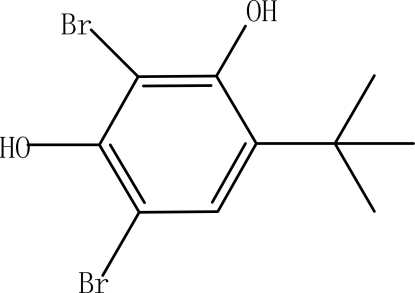


## Experimental

### 

#### Crystal data

C_10_H_12_Br_2_O_2_
*M* *_r_* = 324.02
Tetragonal, 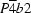

*a* = 11.618 (3) Å
*c* = 17.761 (4) Å
*V* = 2397.4 (9) Å^3^
*Z* = 8
Mo *K*α radiation
μ = 6.74 mm^−1^
*T* = 290 K
0.22 × 0.20 × 0.20 mm

#### Data collection

Oxford Diffraction Xcalibur Eos Gemini diffractometer
Absorption correction: multi-scan (*CrysAlis PRO*; Oxford Diffraction, 2009 ▶) *T*_min_ = 0.319, *T*_max_ = 0.346
4935 measured reflections
1367 independent reflections
775 reflections with *I* > 2σ(*I*)
*R*_int_ = 0.087

#### Refinement

*R*[*F* ^2^ > 2σ(*F* ^2^)] = 0.049
*wR*(*F* ^2^) = 0.059
*S* = 1.08
1367 reflections
132 parameters
H-atom parameters constrained
Δρ_max_ = 0.34 e Å^−3^
Δρ_min_ = −0.35 e Å^−3^

### 

Data collection: *CrysAlis PRO* (Oxford Diffraction, 2009 ▶); cell refinement: *CrysAlis PRO*; data reduction: *CrysAlis PRO*; program(s) used to solve structure: *SHELXS97* (Sheldrick, 2008 ▶); program(s) used to refine structure: *SHELXL97* (Sheldrick, 2008 ▶); molecular graphics: *SHELXTL* (Sheldrick, 2008 ▶); software used to prepare material for publication: *SHELXL97*.

## Supplementary Material

Crystal structure: contains datablock(s) global, I. DOI: 10.1107/S1600536811034866/vm2103sup1.cif

Structure factors: contains datablock(s) I. DOI: 10.1107/S1600536811034866/vm2103Isup2.hkl

Supplementary material file. DOI: 10.1107/S1600536811034866/vm2103Isup3.cml

Additional supplementary materials:  crystallographic information; 3D view; checkCIF report

## Figures and Tables

**Table 1:** Hydrogen-bond geometry (Å, °)

*D*—H⋯*A*	*D*—H	H⋯*A*	*D*⋯*A*	*D*—H⋯*A*
O1—H1⋯O1^i^	0.82	2.67	3.246 (13)	129
O2—H2⋯O2^ii^	0.82	2.36	2.979 (9)	133

Symmetry codes: (i) 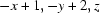; (ii) 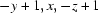.

**Fig. 1.**
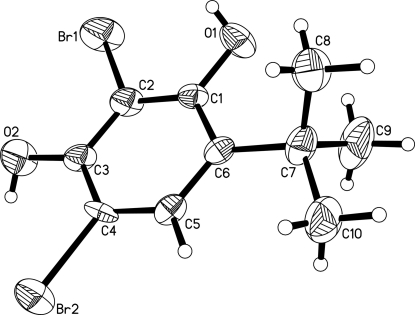
View of the title compound, showing the labeling of the 30% probability ellipsolids.

**Fig. 2.**
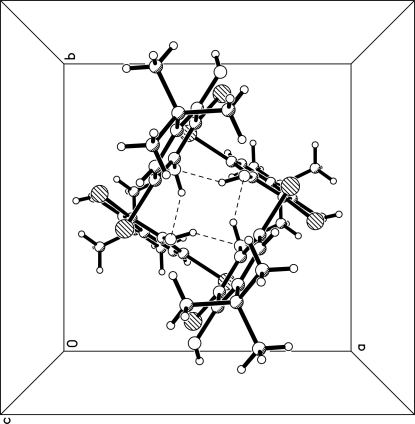
A view of the crystal packing along the c axis. Hydrogen bonds are shown as dashed lines.

## supplementary crystallographic information

### Comment

Bromoarenes have proven to be important and valuable precursors for the synthesis of a wide variety of target compounds by functionalization through cross-coupling reactions, and have been used as key intermediates in the synthesis of a large number of natural products and bioactive materials (Butler & Walker, 1993; Seevers & Counsell, 1982). In this paper, we synthesized the title compound and reported its crystal structure. In the title compound, C—C—C angles within the phenyl ring span a range of 115.7 (7) ° to 122.4 (7) ° with the smallest angle found on the C6 atom bearing the *tert*-butyl substituent, and the largest angle is found for the unsubstituted C5 atom (Fig. 1). In the crystal, molecules are linked by O—H···O hydrogen bonds (Table 1, Fig. 2). In addition, the benzene rings between the adjacent molecules are stacked in a face-to-face orientation with the distance of 3.858 Å, a distance longer than the π–π stacking distances of 3.33 - 3.53 Å reported elsewhere (Zheng *et al.*, 2004), indicating no π–π stacking is observed for this compound.

### Experimental

A mixture of 4,6-di-*tert*-butylbenzene-1,3-diol (111 mg, 0.5 mmol), *p*-toluenesulfonic acid monohydrate (285 mg, 1.5 mmol) and *N*-bromosuccinimide in acetonitrile (2 ml) was heated to reflux for 3 h. Subsequently, the solvent was removed under reduced pressure, and the residue was purified by preparative TLC on silica gel plates (eluent: petroleum ether/EtOAc, 4:1) to give the product as a white solid (282 mg, 87% yield). Colourless single crystals of the title compound suitable for X-ray analysis were obtained by slow evaporation of an acetonitrile solution.

### Refinement

H atoms were generated geometrically and refined as riding atoms with C-H = 0.93Å, O-H = 0.82Å, and U_iso_(H) = 1.2 times U_eq_(C), U_iso_(H) = 1.5 times U_eq_(O)

### Crystal data

**Table:**

C_10_H_12_Br_2_O_2_	*D*_x_ = 1.795 Mg m^−^^3^
*M**_r_* = 324.02	Mo *K*α radiation, λ = 0.71073 Å
Tetragonal, *P*4*b*2	Cell parameters from 1414 reflections
Hall symbol: P -4 -2ab	θ = 3.4–29.0°
*a* = 11.618 (3) Å	µ = 6.74 mm^−^^1^
*c* = 17.761 (4) Å	*T* = 290 K
*V* = 2397.4 (9) Å^3^	Prismatic, colorless
*Z* = 8	0.22 × 0.20 × 0.20 mm
*F*(000) = 1264	

### Data collection

**Table:**

Oxford Diffraction Xcalibur Eos Gemini diffractometer	1367 independent reflections
Radiation source: fine-focus sealed tube	775 reflections with *I* > 2σ(*I*)
graphite	*R*_int_ = 0.087
Detector resolution: 16.2312 pixels mm^-1^	θ_max_ = 26.4°, θ_min_ = 3.4°
ω scans	*h* = −7→14
Absorption correction: multi-scan (*CrysAlis PRO*; Oxford Diffraction, 2009)	*k* = −13→11
*T*_min_ = 0.319, *T*_max_ = 0.346	*l* = −22→11
4935 measured reflections	

### Refinement

**Table:**

Refinement on *F*^2^	Primary atom site location: structure-invariant direct methods
Least-squares matrix: full	Secondary atom site location: difference Fourier map
*R*[*F*^2^ > 2σ(*F*^2^)] = 0.049	Hydrogen site location: inferred from neighbouring sites
*wR*(*F*^2^) = 0.059	H-atom parameters constrained
*S* = 1.08	*w* = 1/[σ^2^(*F*_o_^2^) + (0.0056*P*)^2^] where *P* = (*F*_o_^2^ + 2*F*_c_^2^)/3
1367 reflections	(Δ/σ)_max_ < 0.001
132 parameters	Δρ_max_ = 0.34 e Å^−^^3^
0 restraints	Δρ_min_ = −0.35 e Å^−^^3^

### Special details

**Table:**

Experimental. CrysAlisPro (Oxford Diffraction, 2009) Empirical absorption correction using spherical harmonics, implemented in SCALE3 ABSPACK scaling algorithm.
Geometry. All e.s.d.'s (except the e.s.d. in the dihedral angle between two l.s. planes) are estimated using the full covariance matrix. The cell e.s.d.'s are taken into account individually in the estimation of e.s.d.'s in distances, angles and torsion angles; correlations between e.s.d.'s in cell parameters are only used when they are defined by crystal symmetry. An approximate (isotropic) treatment of cell e.s.d.'s is used for estimating e.s.d.'s involving l.s. planes.
Refinement. Refinement of *F*^2^ against ALL reflections. The weighted *R*-factor *wR* and goodness of fit *S* are based on *F*^2^, conventional *R*-factors *R* are based on *F*, with *F* set to zero for negative *F*^2^. The threshold expression of *F*^2^ > σ(*F*^2^) is used only for calculating *R*-factors(gt) *etc*. and is not relevant to the choice of reflections for refinement. *R*-factors based on *F*^2^ are statistically about twice as large as those based on *F*, and *R*- factors based on ALL data will be even larger.

### Fractional atomic coordinates and isotropic or equivalent isotropic displacement parameters (Å^2^)

**Table:**

	*x*	*y*	*z*	*U*_iso_*/*U*_eq_	
Br1	0.54152 (12)	0.82654 (10)	0.57359 (5)	0.1038 (5)	
Br2	0.26935 (9)	0.44073 (9)	0.65318 (6)	0.0780 (4)	
O1	0.5347 (6)	0.8647 (5)	0.7424 (3)	0.076 (2)	
H1	0.5215	0.9175	0.7131	0.114*	
O2	0.4052 (6)	0.6107 (6)	0.5499 (2)	0.085 (2)	
H2	0.4250	0.5434	0.5444	0.128*	
C1	0.4723 (7)	0.7689 (7)	0.7214 (4)	0.045 (2)	
C2	0.4666 (7)	0.7357 (7)	0.6471 (4)	0.058 (2)	
C3	0.4069 (7)	0.6383 (8)	0.6250 (4)	0.055 (3)	
C4	0.3557 (7)	0.5756 (6)	0.6803 (5)	0.050 (2)	
C5	0.3603 (7)	0.6096 (7)	0.7559 (4)	0.047 (2)	
H5	0.3220	0.5655	0.7917	0.056*	
C6	0.4188 (7)	0.7050 (7)	0.7788 (4)	0.046 (2)	
C7	0.4255 (8)	0.7422 (9)	0.8617 (4)	0.065 (3)	
C8	0.3682 (9)	0.8603 (8)	0.8714 (4)	0.090 (4)	
H8A	0.2916	0.8579	0.8512	0.134*	
H8B	0.4123	0.9176	0.8452	0.134*	
H8C	0.3648	0.8794	0.9240	0.134*	
C9	0.5530 (9)	0.7473 (11)	0.8882 (5)	0.121 (4)	
H9A	0.5558	0.7698	0.9402	0.182*	
H9B	0.5943	0.8024	0.8583	0.182*	
H9C	0.5877	0.6728	0.8825	0.182*	
C10	0.3623 (9)	0.6572 (8)	0.9131 (4)	0.093 (3)	
H10A	0.3963	0.5822	0.9082	0.139*	
H10B	0.2827	0.6536	0.8990	0.139*	
H10C	0.3684	0.6825	0.9644	0.139*	

### Atomic displacement parameters (Å^2^)

**Table:**

	*U*^11^	*U*^22^	*U*^33^	*U*^12^	*U*^13^	*U*^23^
Br1	0.1535 (13)	0.0852 (10)	0.0726 (6)	−0.0324 (7)	0.0515 (8)	0.0131 (6)
Br2	0.0772 (8)	0.0694 (8)	0.0875 (7)	−0.0210 (6)	0.0110 (7)	−0.0109 (6)
O1	0.080 (6)	0.068 (5)	0.079 (4)	−0.032 (5)	0.013 (3)	−0.007 (3)
O2	0.130 (7)	0.084 (6)	0.041 (3)	−0.009 (5)	0.028 (3)	−0.012 (3)
C1	0.049 (6)	0.037 (6)	0.050 (4)	−0.005 (4)	0.010 (5)	0.001 (4)
C2	0.070 (7)	0.054 (6)	0.049 (5)	−0.013 (5)	0.023 (5)	0.002 (5)
C3	0.070 (7)	0.053 (7)	0.043 (5)	−0.009 (5)	0.019 (5)	0.011 (5)
C4	0.047 (6)	0.026 (5)	0.076 (6)	−0.007 (4)	0.009 (5)	−0.004 (4)
C5	0.038 (6)	0.060 (7)	0.041 (5)	0.003 (5)	0.011 (4)	0.013 (5)
C6	0.047 (6)	0.048 (6)	0.044 (4)	−0.002 (5)	0.010 (4)	0.012 (5)
C7	0.067 (7)	0.084 (8)	0.046 (5)	−0.005 (6)	0.000 (5)	0.007 (5)
C8	0.120 (11)	0.094 (10)	0.055 (6)	0.009 (7)	0.022 (6)	−0.011 (5)
C9	0.108 (11)	0.162 (13)	0.094 (7)	−0.020 (8)	−0.047 (7)	0.023 (7)
C10	0.112 (11)	0.112 (10)	0.054 (6)	−0.013 (7)	0.006 (6)	0.009 (6)

### Geometric parameters (Å, °)

**Table:**

Br1—C2	1.891 (7)	C6—C7	1.536 (10)
Br2—C4	1.922 (7)	C7—C10	1.532 (11)
O1—C1	1.379 (8)	C7—C8	1.536 (11)
O1—H1	0.8200	C7—C9	1.556 (11)
O2—C3	1.373 (8)	C8—H8A	0.9600
O2—H2	0.8200	C8—H8B	0.9600
C1—C2	1.377 (9)	C8—H8C	0.9600
C1—C6	1.406 (9)	C9—H9A	0.9600
C2—C3	1.384 (10)	C9—H9B	0.9600
C3—C4	1.360 (10)	C9—H9C	0.9600
C4—C5	1.400 (9)	C10—H10A	0.9600
C5—C6	1.362 (10)	C10—H10B	0.9600
C5—H5	0.9300	C10—H10C	0.9600
			
C1—O1—H1	109.5	C6—C7—C8	109.7 (6)
C3—O2—H2	109.5	C10—C7—C9	107.5 (7)
C2—C1—O1	120.7 (7)	C6—C7—C9	110.4 (7)
C2—C1—C6	121.7 (7)	C8—C7—C9	110.2 (10)
O1—C1—C6	117.5 (7)	C7—C8—H8A	109.5
C1—C2—C3	121.6 (7)	C7—C8—H8B	109.5
C1—C2—Br1	118.9 (6)	H8A—C8—H8B	109.5
C3—C2—Br1	119.5 (5)	C7—C8—H8C	109.5
C4—C3—O2	124.7 (8)	H8A—C8—H8C	109.5
C4—C3—C2	117.0 (7)	H8B—C8—H8C	109.5
O2—C3—C2	118.3 (7)	C7—C9—H9A	109.5
C3—C4—C5	121.6 (7)	C7—C9—H9B	109.5
C3—C4—Br2	119.0 (6)	H9A—C9—H9B	109.5
C5—C4—Br2	119.4 (6)	C7—C9—H9C	109.5
C6—C5—C4	122.4 (7)	H9A—C9—H9C	109.5
C6—C5—H5	118.8	H9B—C9—H9C	109.5
C4—C5—H5	118.8	C7—C10—H10A	109.5
C5—C6—C1	115.7 (7)	C7—C10—H10B	109.5
C5—C6—C7	122.7 (7)	H10A—C10—H10B	109.5
C1—C6—C7	121.6 (8)	C7—C10—H10C	109.5
C10—C7—C6	111.5 (8)	H10A—C10—H10C	109.5
C10—C7—C8	107.5 (7)	H10B—C10—H10C	109.5
			
O1—C1—C2—C3	178.4 (9)	Br2—C4—C5—C6	−179.3 (7)
C6—C1—C2—C3	0.7 (14)	C4—C5—C6—C1	1.4 (13)
O1—C1—C2—Br1	−2.1 (12)	C4—C5—C6—C7	−179.3 (7)
C6—C1—C2—Br1	−179.8 (7)	C2—C1—C6—C5	−0.8 (13)
C1—C2—C3—C4	−1.1 (13)	O1—C1—C6—C5	−178.6 (8)
Br1—C2—C3—C4	179.4 (6)	C2—C1—C6—C7	179.9 (8)
C1—C2—C3—O2	−180.0 (8)	O1—C1—C6—C7	2.1 (12)
Br1—C2—C3—O2	0.5 (12)	C5—C6—C7—C10	1.8 (12)
O2—C3—C4—C5	−179.5 (7)	C1—C6—C7—C10	−178.9 (8)
C2—C3—C4—C5	1.7 (12)	C5—C6—C7—C8	−117.2 (10)
O2—C3—C4—Br2	−2.2 (12)	C1—C6—C7—C8	62.1 (11)
C2—C3—C4—Br2	179.0 (7)	C5—C6—C7—C9	121.2 (10)
C3—C4—C5—C6	−2.0 (13)	C1—C6—C7—C9	−59.5 (12)

### Hydrogen-bond geometry (Å, °)

**Table:**

*D*—H···*A*	*D*—H	H···*A*	*D*···*A*	*D*—H···*A*
O1—H1···O1^i^	0.82	2.67	3.246 (13)	129.
O2—H2···O2^ii^	0.82	2.36	2.979 (9)	133.

Symmetry codes: (i) −*x*+1, −*y*+2, *z*; (ii) −*y*+1, *x*, −*z*+1.
